# Analyses of Methylomes Derived from Meso-American Common Bean (*Phaseolus vulgaris* L.) Using MeDIP-Seq and Whole Genome Sodium Bisulfite-Sequencing

**DOI:** 10.3389/fpls.2016.00447

**Published:** 2016-04-26

**Authors:** Mollee Crampton, Venkateswara R. Sripathi, Khwaja Hossain, Venu Kalavacharla

**Affiliations:** ^1^Molecular Genetics and Epigenomics Laboratory, Delaware State University, DoverDE, USA; ^2^Division of Science and Mathematics, Mayville State University, MayvilleND, USA; ^3^Center for Integrated Biological and Environmental Research, Delaware State University, DoverDE, USA

**Keywords:** *Phaseolus vulgaris*, common bean, methylome, whole genome bisulfite-sequencing, MeDIP-Seq

## Abstract

Common bean (*Phaseolus vulgaris* L.) is economically important for its high protein, fiber, and micronutrient contents, with a relatively small genome size of ∼587 Mb. Common bean is genetically diverse with two major gene pools, Meso-American and Andean. The phenotypic variability within common bean is partly attributed to the genetic diversity and epigenetic changes that are largely influenced by environmental factors. It is well established that an important epigenetic regulator of gene expression is DNA methylation. Here, we present results generated from two high-throughput sequencing technologies, methylated DNA immunoprecipitation-sequencing (MeDIP-seq) and whole genome bisulfite-sequencing (BS-Seq). Our analyses revealed that this Meso-American common bean displays similar methylation patterns as other previously published plant methylomes, with CG ∼50%, CHG ∼30%, and CHH ∼2.7% methylation, however, these differ from the common bean reference methylome of Andean origin. We identified higher CG methylation levels in both promoter and genic regions than CHG and CHH contexts. Moreover, we found relatively higher CG methylation levels in genes than in promoters. Conversely, the CHG and CHH methylation levels were highest in promoters than in genes. This is the first genome-wide DNA methylation profiling study in a Meso-American common bean cultivar (“Sierra”) using NGS approaches. Our long-term goal is to generate genome-wide epigenomic maps in common bean focusing on chromatin accessibility, histone modifications, and DNA methylation.

## Introduction

Common bean (*Phaseolus vulgaris*) is the most widely consumed legume, as it is high in protein, fiber, and essential nutrients, while low in glycemic index (Mitchell et al., 2009). The haploid genome size of common bean is about 587 Mb (Schmutz et al., 2014). Bean domestication is assumed to be a complex process that involved two distinct gene pools (Meso-American and Andean) and repeated selection of desirable traits within the gene pool (Bitocchi and Nanni, 2012). Common bean is consumed widely across the world due to its affordability and health benefits (Gepts et al., 2008). Food legumes such as common bean are an inherently rich source of vitamins and minerals, including B vitamins (particularly B1/thiamine and B9/folic acid), iron, zinc, calcium and magnesium (Akond et al., 2011; Beebe et al., 2013; Câmara et al., 2013; Petry et al., 2015). Hence, common bean is considered as a viable target for biofortification (Blair, 2013). In order to effectively modulate genetic architecture and develop micronutrient-enriched crops, understanding the genes and their regulatory mechanisms involved in physiological processes is important. Though several regulatory mechanisms have been identified, DNA methylation, chromatin remodeling, histone acetylation, histone methylation, and gene silencing are of paramount importance.

DNA methylation is a covalent, heritable epigenetic modification that plays a significant role in gene expression, tissue specialization, and transposon inactivation (Zhang et al., 2006; Verhoeven et al., 2010). In plants, cytosine methylation occurs in both symmetrical (CG and CHG) and asymmetrical (CHH) contexts, while descending order of the extent of methylation in these contexts is CG, CHG, and CHH, where H indicates A, C, or T (Lister and Ecker, 2009; Henderson et al., 2010; Saze et al., 2012). DNA methylation occurs at higher levels in heterochromatin when compared to euchromatin (Madlung and Comai, 2004; Song et al., 2013). Genome-wide DNA methylation and gene expression studies revealed that gene-body methylation is conserved across species (Feng et al., 2010), among constitutively expressed genes (Zhang et al., 2006), and more specifically, CG methylation is often linked to increased gene expression (Miura et al., 2009; Wang et al., 2014; Kim et al., 2015). Conversely, promoter DNA methylation in plants is largely associated with transcriptional repression (Li et al., 2008) and is highly tissue-specific in nature (Zhang et al., 2006; Song et al., 2013). In soybean (dicot) and rice (monocot), it has been reported that promoter hypomethylation resulted in increased gene expression of flanking genes (Li et al., 2008; Song et al., 2013).

There are several methods for determining the presence and location of methylated cytosines in the genome, which include methylation-sensitive restriction enzyme sequencing (MRE-seq), affinity purification, and whole genome bisulfite-sequencing (BS-Seq). This study used one of the three affinity purification methods, i.e., methylated DNA immunoprecipitation sequencing (MeDIP-seq) and BS-Seq. MeDIP-Seq utilizes anti-5-mC antibody and generates ∼10^7^ reads while BS-Seq being a “gold standard” of methylation sequencing, generates ∼10^8^ methylated cytosines at single nucleotide resolution. This technique converts unmethylated cytosines to uracil by deamination, while methylated cytosines remain as cytosines (Lister and Ecker, 2009; Laird, 2010; Kim et al., 2014). A genome-wide DNA methylation map has been reported in Andean common bean (Kim et al., 2015) but it is not available in Meso-American common bean. The previous methylome study compared the methylation in Andean common bean leaf to soybean leaf, stripped root, and root hair methylomes (Kim et al., 2015). Screening the methylomes of Meso-American common bean (*P. vulgaris*) using high-throughput approaches such as BS-Seq and MeDIP-Seq for genome-wide DNA methylation may aid in understanding key regulatory mechanisms linked to important biological processes.

## Results

Whole genome bisulfite-sequencing (BS-Seq) is widely used to study DNA methylation at the whole genome level while MeDIP-Seq is used to detect high DNA methylation in low-density CG/CHG/CHH areas or moderate methylation levels in high-density CG/CHG/CHH regions. To understand the DNA methylation levels in the three contexts (CG, CHG, and CHH), we constructed two MeDIP-Seq libraries (one input control that served as no antibody sample and one immunoprecipitated sample) and one BS-Seq library using tissue collected from three sets of bean plants from the Meso-American common bean cultivar, Sierra, grown at three different times (three biological replicates grown in triplicate) which were pooled and sequenced on the Illumina/HiSeq-2500 platform. A similar replicate pooling approach has been used in other epigenomic studies that utilized next generation sequencing technologies (Taylor et al., 2007; Brinkman et al., 2012; Statham et al., 2012). The libraries generated in this study were labeled as Sierra_Input, Sierra_MeDIP and Sierra_BS (**Table 1**). The input control (Sierra_Input) was used for comparing against immunoprecipitated DNA (Sierra_MeDIP) during MeDIP-Seq analysis to identify differentially methylated regions (DMRs).

**Table 1:** Reads and mapping ratios for each sample.

Sample name	Method	Total read #	Mapped read #	Mapping ratio (%)
Sierra_BS	BS-Seq	214,016,819	61,362,998	28.67
Sierra_Input	INPUT	22,509,873	8,897,764	39.53
Sierra_MeDIP	MeDIP-seq	23,709,423	7,173,104	30.25


### Data Collection and Preprocessing

Deep sequencing of three methylome libraries in common bean resulted in ∼260 million-50 bp Illumina reads (**Table 1**). Of these, ∼214 million reads were from BS-Seq and the rest (∼47 million reads) were from MeDIP-Seq. Among MeDIP-Seq reads, ∼22.5 million reads were from input control and ∼23.7 million were methylated DNA immunoprecipitated reads. The raw reads obtained from both methodologies were trimmed, filtered, and high quality reads collected were aligned to the reference *P. vulgaris* genome, G19833 (Schmutz et al., 2014) available at Phytozome (V1.0, accessed October 2014). In total, ∼61.4 (28.67%), ∼8.9 (39.53%) and ∼7.2 (30.25%) million BS-Seq, MeDIP-Seq input control, and MeDIP-Seq immunoprecipitated reads were mapped to the reference genome (G19833). Of these, uniquely mapped reads with ≤2 mis-matches were further used in analysis.

### Bisulfite-Sequencing (BS-Seq)

**FIGURE 1:**
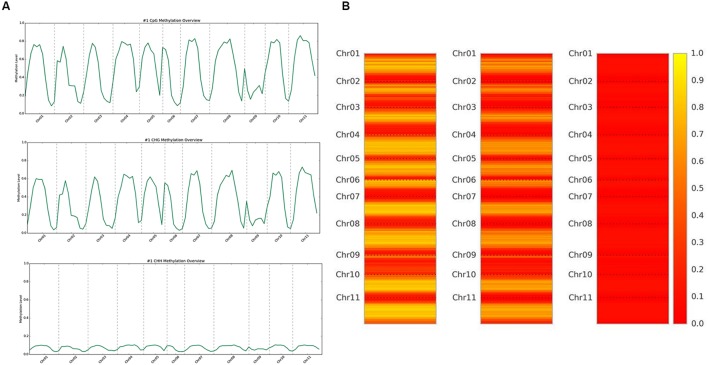
**(A)** Whole genome overview of DNA methylation levels, by context. The top is CG methylation, middle is CHG methylation, and bottom is CHH methylation levels across all 11 chromosomes of common bean. The scale ranges from 0.0 (no methylation) up to 1.0 (100% methylation). **(B)** Heat map representation of methylation levels, by context. The left is CG methylation, the middle is CHG methylation, and the right is CHH methylation levels across all 11 chromosomes of common bean. The scale ranges from 0.0/red (no methylation) up to 1.0/yellow (100% methylation).

**FIGURE 2:**
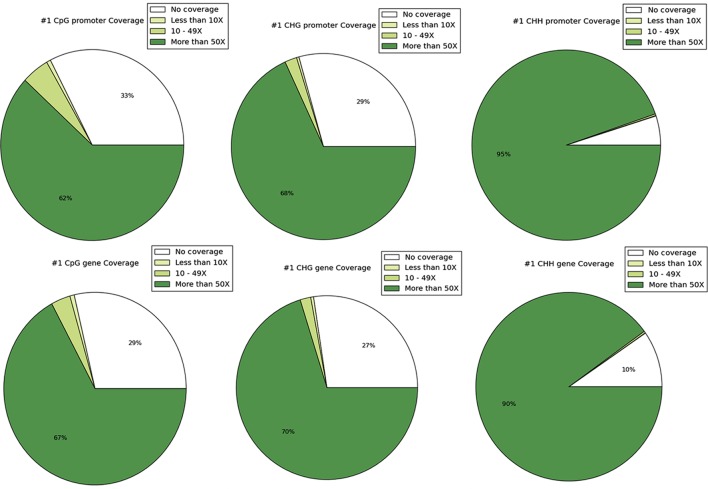
**The coverage of sites is shown and depicted in the categories of 0 coverage, <10× coverage, 10–49× coverage, and ≥50 coverage.** The top row shows coverage by context in promoters and the bottom row shows coverage by context in genes.

DNA methylation ratios from BS-Seq were evaluated for CG, CHG, and CHH contexts. The increasing order of methylation levels in the three contexts were CHH followed by CHG and CG. Though the methylation patterns for CG and CHG contexts were similar, the overall levels of CHG methylation were relatively lower than CG methylation. The genome-wide DNA methylation levels for each context are shown (**Figures 1A,B**). The least methylated context, CHH was consistently very low across all 11 common bean chromosomes. In addition to the overall methylation across the three contexts, we also investigated the methylated cytosines in promoter and genic regions. The promoter and gene coverage between CG (67%, 71%) and CHG (71%, 73%) contexts varied when compared to CHH coverage (95%, 90%) context. While the majority of the reads covered in all contexts were more than 50-fold at each cytosine site, some sites were not covered (**Figure 2**). For each context, the overall read coverage (number of reads per cytosine) was determined as either 0 (no coverage), 1–4, 5–10, 11–20, and >20 reads per cytosine (**Supplementary Figure S2**). The majority of the covered reads was more than 11 reads per cytosine and this trend was consistent across the three contexts of methylation measured by BS-Seq.

**FIGURE 3:**
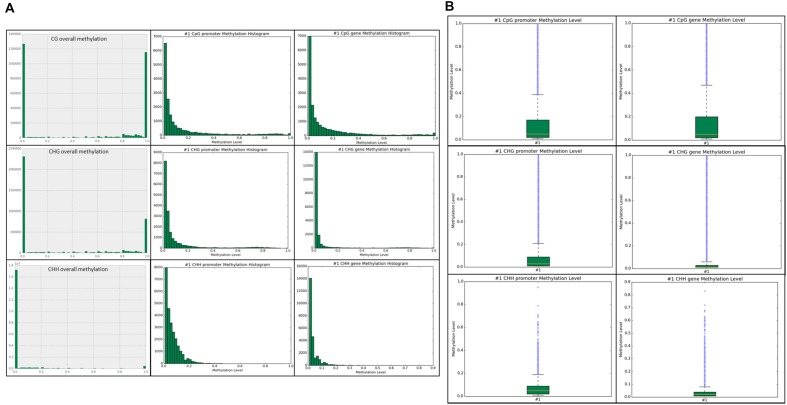
**(A)** Unique sites detected for each context sequence is shown. The top row is the CG overall methylation throughout the genome, followed by CG promoter methylation, and CG gene methylation. The middle row is CHG overall, promoter, and gene methylation. The bottom row is CHH overall, promoter, and gene methylation. The *x*-axis is methylation level in 0.1 intervals, ranging from 0.0–1.0. The *y*-axis is the frequency of the particular methylation level in that context in the associated genomic feature. **(B)** Boxplots of promoter and genic methylation levels are shown on a scale of 0.0–1.0.

Methylation ratios and their frequencies throughout the genome were analyzed in each context. Within the CG context, ∼2.65 million total sites and ∼1.25 million methylated sites ≥ 0.8 (∼50%) were identified across the genome. In the CHG context, ∼3.3 million total sites and ∼one million methylated sites (∼30%) were identified. The most prominent context was CHH and we identified approximately 18 million CHH sites in the genome, but only ∼500,000 sites were methylated (2.7%) with the vast majority of sites unmethylated. Our results show that most of the methylation and highly methylated regions are found outside of the promoter and genic regions (intragenic) in all three contexts (**Figure 3A**). Methylation levels for the annotated promoter and genic regions are presented in box plots for each context (**Figure 3B**). Higher CG methylation was found in genic regions when compared to the promoters. Conversely, comparatively higher CHG and CHH methylation was observed in promoter regions than genic regions. However, the overall CG methylation was highest among the three contexts.

### Methylated DNA Immunoprecipitation (MeDIP-Seq)

To understand the methylome of common bean, assessing the peak density and peak shape associated with DNA methylation is necessary. Peaks can be called by mapping the reads to the genome to reveal the loci of selectively methylated DNA. Several peak callers are currently available to identify the global pattern of these modified sites by comparing the antibody tagged sample with its background (input, no antibody). The shapes are based on genomic coverage of the input control, the coverage of the DNA that was not subjugated to immunoprecipitation with an anti-5mC antibody, versus the immunoprecipitated DNA. The increased coverage is displayed as a “peak,” which implies that there is more coverage, due to the enrichment of DNA containing methylated cytosines (**Supplementary Figure S1**). Peak assessment is very important in minimizing the false discovery rate and developing a gold-standard common bean methylome. A correlation between peak density and chromosomal length has been proposed in mammals (Hughes et al., 2010). We estimated the methylation peak density (peak/Mb) based on chromosome length and total number of peaks identified per chromosome. MeDIP-Seq signatures per chromosome, total peaks across the genome, peaks found only in promoter regions, and peaks identified in genic locations are presented (**Table 2**). The majority (93.2%) of the peaks identified from MeDIP-Seq analysis were in non-promoter and non-genic regions. The peaks obtained from MeDIP-Seq were compared against the methylation sites from BS-Seq data to corroborate the peaks identified in likely methylated regions and to compare the two methylome sequencing work flows. The top 20 most significant (*p* < 0.05) MeDIP-Seq peaks were compared to CG and CHG weighted methylation determined from BS-Seq to identify that all of these methylated cytosine regions possess methylation ratios over 0.5 in CG and/or CHG contexts (**Table 3**).

**Table 2:** Common bean chromosomal length, reads, signatures, and total and annotated peaks from MeDIP-Seq data.

Reference	Chrom length (Mbp)	Reads	Signatures	Total peaks	Peaks in promoter	Peaks in genes
Chr01	52.2	1,889,081	35,436	3,261	130	80
Chr02	49	1,413,718	27,421	2,977	103	99
Chr03	52.3	1,711,966	31,614	3,037	128	77
Chr04	45.9	1,916,912	31,458	2,943	92	65
Chr05	40.8	1,548,871	26,404	2,572	109	82
Chr06	32	967,065	17,434	1,544	81	72
Chr07	51.8	2,066,300	34,473	3,598	130	83
Chr08	59.7	2,877,212	34,359	3,774	137	109
Chr09	37.5	1,194,008	16,610	1,919	97	71
Chr10	43.3	1,986,809	29,905	3,091	105	101
Chr11	50.4	2,473,345	34,568	3,740	153	104
Total	514.9	20,045,287	319,682	32,456	1,265	943


**Table 3:** The top 20 most significant/highest peak shape scores from the MeDIP-Seq data are shown.

Length	Peak shape score	*P*-value	5′ gene	5′ distance	3′ gene	3′ distance	CG	CHG
243	15.03	2.30E–51			Phvul.008G000100	0	0.897252	N/A
237	14.09	2.24E–45			Phvul.008G000100	95707	0.932642	0.892308
231	13.66	8.72E–43			Phvul.008G000100	112068	0.686242	0.679463
204	13.38	3.70E–41	Phvul.004G046900	2257	Phvul.004G047000	4936	0.949129	N/A
210	13.29	1.35E–40	Phvul.008G293200	8476			0.850638	N/A
246	12.87	3.41E–38			Phvul.008G000100	100985	0.857233	0.726482
206	12.76	1.29E–37	Phvul.008G293200	4372			0.763093	0.166279
226	12.71	2.57E–37	Phvul.009G144700	60451	Phvul.009G144800	28262	0.897214	0.823301
242	12.18	2.05E–34	Phvul.008G293200	6471			0.9447	1
209	12.03	1.23E–33	Phvul.008G293200	8724			0.92825	N/A
235	11.95	3.34E–33			Phvul.008G000100	108787	0.842105	0.88024
212	11.91	5.27E–33			Phvul.008G000100	113332	0.807415	0.734861
221	11.43	1.41E–30	Phvul.009G144700	50026	Phvul.009G144800	38692	N/A	0.775591
210	11.36	3.29E–30			Phvul.008G000100	110552	0.925373	0.892
214	11.31	5.68E–30			Phvul.008G000100	105211	0.832864	0.814815
220	11.27	8.84E–30	Phvul.009G144700	51399	Phvul.009G144800	37320	0.5	0.779538
204	11.01	1.69E–28			Phvul.008G000100	109425	N/A	0.892105
206	10.83	1.26E–27	Phvul.009G144700	58862	Phvul.009G144800	29871	0.839901	0.689939
274	10.76	2.59E–27	Phvul.009G144700	53308	Phvul.009G144800	35357	0.850394	0.730228
223	10.35	2.11E–25	Phvul.008G293200	2479			0.961965	N/A


*Also shown are peak locations in relation to 5′ and 3′ genes. CG and CHG methylation ratios were determined from BS-Seq data in the corresponding peak locations.*

## Discussion

DNA methylation is a heritable epigenetic mark that controls a wide variety of physiological processes (Boyko et al., 2007). Though DNA methylation has been reported in many eukaryotic organisms, the contexts and levels of DNA methylation vary significantly between plants and animals. In animals, the symmetric context, CG, is most prominent while non-CG (CHG and CHH) methylation is less frequent (Stroud et al., 2013b). In plants, DNA is primarily methylated in the CG context while non-CG contexts (CHG and CHH) are also abundant. The reports on CG and non-CG methylation in plants are increasingly evident. For example, in *Arabidopsis* both CG and non-CG methylation are functional (Law and Jacobsen, 2010) and the roles of chromomethyltransferase (CMT) and domains rearranged methyltransferase (DRM) proteins in non-CG methylation has been established (Cao et al., 2003; Stroud et al., 2013b).

MeDIP-Seq yields a resolution of ∼100–200 bp while single base resolution methylome maps can be obtained by both conventional Sanger sequencing of short BS-treated fragments and whole genome bisulfite-sequencing (Taiwo et al., 2012; Stevens et al., 2013). In common bean, we generated 10-fold more reads using BS-Seq when compared to MeDIP-Seq, as previously reported (Laird, 2010). The relatively lower number of reads in MeDIP-Seq compared to BS-Seq is due to the inherent difference between the techniques (Laird, 2010). However, MeDIP-Seq analysis identified densely methylated regions in the genome while BS-Seq analysis estimated overall methylation rates in the genome.

Currently, few reports of BS-Seq are available in plants, which are mostly model species and some are presented (**Table 4**). Epigenome reports in legume crops are scantly available. Recently, single nucleotide resolution reference methylomes in soybean and Andean common bean have been constructed using BS-Seq in order to understand epigenetic variation in legume crops (Kim et al., 2015). Here we generated single nucleotide resolution (BS-Seq) and 150 bp resolution (MeDIP-Seq) maps of DNA methylation to understand genome-wide DNA methylation in Meso-American common bean. Further, we focused our analyses in estimating the overall methylation levels, context-specific methylation patterns, and promoter versus gene body methylation patterns. This study represents the first genome-wide DNA methylation report in the important rust-resistant Meso-American common bean cultivar, Sierra.

**Table 4:** Summary of BS-Seq studies in plants.

Species	Reference
*Amborella trichopoda*	Albert et al., 2013
*Arabidopsis lyrata*	Seymour et al., 2014
*Arabidopsis thaliana*	Cokus et al., 2008; Hseih et al., 2009; Lister et al., 2009; Greaves et al., 2012; Shen et al., 2012; Schmitz et al., 2013
*Brachypodium distachyon*	Takuno and Gaut, 2012
*Capsella rubella*	Seymour et al., 2014
*Glycine max*	Schmitz et al., 2013; Song et al., 2013; Kim et al., 2015
*Oryza sativa*	Zemach et al., 2010; Chodavarapu et al., 2012; Li et al., 2012; Stroud et al., 2013a
*Phaseolus vulgaris*	Kim et al., 2015
*Solanum lycopersicum*	Stroud et al., 2013b
*Zea mays*	Eichten et al., 2013; Gent et al., 2013; Regulski et al., 2013; West et al., 2014


### Genome-Wide Methylation Levels

We aligned the sequenced cytosines collected from MeDIP-Seq and BS-Seq to the common bean reference genome (G19833) to identify genome-wide methylation, site-specific methylation and annotated the methylated peaks. In total, 3.0–3.5 million cytosines were found to be methylated across the genome covering all three contexts of DNA methylation, thus accounting for <1% of the total DNA bases in the genome. A total number of 32,456 significant (>1.5-fold-enrichment) peaks were identified in common bean. The average DNA methylation peak density identified was ∼63 peaks/1 Mb of DNA. Based on the average peak density, lower methylation levels were identified in chromosomes, Chr02, Chr03, Chr06, and Chr09. Conversely, higher methylation levels were noted in chromosomes, Chr07, Chr10, and Chr11 (**Table 2**). Similar patterns in methylation saturation between chromosomes were highlighted by using chromosome-based methylation distribution heat maps (**Figure 1B**). Genome-wide methylation identified in this study was over 37.8% in CG and CHG contexts, which is in accordance with the other reports in *Arabidopsis*, rice and soybean (Cokus et al., 2008; Li et al., 2012; Song et al., 2013).

### Context-Specific Methylation Patterns

In common bean, we generated methylation histograms in order to show the methylation ratios (0.0–1.0) for the cytosine sites in each context within the genome and for the annotated genomic features (**Figure 2**). The majority of the overall methylation ratios for hemi-methylated or fully methylated sites ranged between 0.8 and 1.0 in the CG context, which is in accordance with CG methylation in *Arabidopsis*. The distribution of methylation ratios in the CHG context for the hemi-methylated or fully methylated sites ranged between mainly 0.6-1.0 while in *Arabidopsis* the ratios reported were between 0.2 and1.0 (Cokus et al., 2008). Within bean, the CG and CHG distributions were similar except for fewer fully methylated sites (ratio = 1.0) in the CHG context. The CHH methylation ratios were predominately <10% in *Arabidopsis* (Cokus et al., 2008; Feng et al., 2010), maize (Eichten et al., 2013), and many other eukaryotic organisms (Feng et al., 2010) which is consistent with the present findings in common bean. As CHH methylation is commonly found in repetitive and transposable elements, it varies significantly more between tissues and even among biological replicates than CG and CHG methylation (Song et al., 2013; Kim et al., 2015).

In addition to the methylation ratios, the overall methylation levels in our study estimated for CG, CHG and CHH contexts were ∼50, ∼30, and ∼2.7%, respectively. In *Arabidopsis*, the methylation levels specific to each context (∼24% in CG, 6.7% in CHG, and 1.7% in CHH contexts) were reported (Cokus et al., 2008). A similar study which screened eight eukaryotic species identified ∼22% of CGs, ∼6.0% of CHGs, and ∼2% of CHHs as methylated in *Arabidopsis* (Feng et al., 2010). In poplar, the methylation level, 41.9%, 20.9%, and 3.25% were reported for CG, CHG, and CHH contexts, respectively (Feng et al., 2010). Recently, among legumes, the methylation percentage for CG (74% and 64%), CHG (62% and 48%) and CHH (21% and 4%) contexts have been reported in common bean and soybean leaves, respectively (Kim et al., 2015). Other leaf methylation levels reported in soybean include, 63% in CG, 44% in CHG, and 5.9% in CHH contexts (Song et al., 2013). Our results were more similar to the methylation levels for each context in poplar than in soybean. This is likely attributed to the fact that bean and poplar have a similar genome size when compared to soybean. Moreover, soybean has experienced a whole-genome duplication after diverging from common bean. Interestingly, our results slightly deviated from the earlier reports in common bean for all the three contexts (Kim et al., 2015). The deviation in the methylation ratios in this study may be attributed to the genotype-specific DNA methylation variation (Meso-American vs Andean), differences in leaf stages (primary vs. trifoliate) and collection time-points (14-day-old vs. 18-day-old) used while analyzing the bean methylome.

### Promoter and Gene-Body Methylation

Promoter methylation and genic methylation have been implicated in diverse regulatory roles. Generally, cytosine methylation in promoter regions results in transcriptional repression in plants. A single methylated cytosine in a promoter region can significantly affect the expression level of the corresponding gene. About 16.6% of methylated single CGs identified in the promoter adjacent to transcriptional start sites decreased gene expression in humans (Medvedeva et al., 2014). CG and CHG promoter methylation typically causes repression of adjacent genes, as reported in *Arabidopsis*, maize, and soybean (Zhang et al., 2006; Song et al., 2013; West et al., 2014). The role of gene-body methylation in controlling alternative promoters and gene splicing has been implicated in mammals (Lou et al., 2014). Gene-body methylation is considered as an evolutionary consequence and it is conserved among plant orthologs (Takuno and Gaut, 2012). With this background we attempted to understand the promoter and genic methylation in common bean.

After annotating the methylated sites for genomic features, we identified higher CG methylation levels in both promoter and genic regions than CHG and CHH contexts (**Figure 3A**). Moreover, we found relatively higher CG methylation levels in genes than in promoters. Conversely, the CHG and CHH methylation levels were highest in promoters than in genes, which is consistent with the findings in *Arabidopsis* (Cokus et al., 2008). We further confirmed that the CG methylation levels are higher in the annotated genomic features (**Figure 3B**). The most significant MeDIP-Seq peaks were found to correlate with high levels of both CG and CHG methylation (**Table 3**). A study in *Arabidopsis* revealed that important functional genes evolved slowly when they contained methylation in the gene-body (Takuno and Gaut, 2012). Another study in *Arabidopsis* found high levels of methylation in gene-bodies (Zhang et al., 2006). Further, it was found that single-copy genes in soybean and common bean were methylated more frequently than duplicated genes (Kim et al., 2015). This suggests that gene-body methylation may have an important evolutionary role, possibly in protecting genes from mutation. DNA methylation has been implicated in physiological and developmental processes in plants. A study conducted in cotton suggested the role of CHH methylation in time-of-day and time-of-year memory (Jin et al., 2013). The fluctuation in DNA methylation pattern in response to different time intervals (Jin et al., 2013) and DNA methylation diversity between genotypes have been proposed in cotton (Osabe et al., 2014). Similarly, we presume differences in DNA methylation found between G19833 (Kim et al., 2015) and Sierra (current study) in common bean is partially due to the fact that two genotypes are derived from different gene pools.

### Comparison of BS-Seq and MeDIP-Seq

As BS-Seq and MeDIP-Seq employ different mechanisms for determining methylated cytosines, the bioinformatic output of each method is also different. In the current study, BS-Seq (∼214 million reads) generated approximately 10-fold more reads than that of the immunoprecipitated reads via MeDIP-Seq (∼22.5 million reads). The cost per sample was about a twofold difference, BS-Seq being the more expensive option. Due to the expense, targeted bisulfite conversion, and sequencing is often chosen for specific genes or regions. However, this option gives much less information than both high-throughput sequencing technologies MeDIP-Seq and BS-Seq.

**FIGURE 4:**
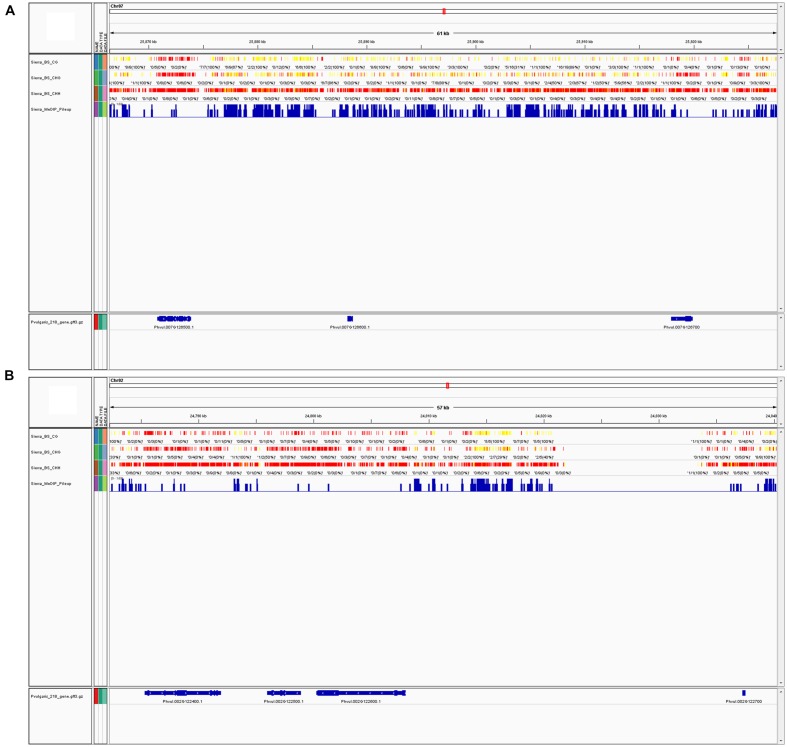
**(A)** MeDIP-Seq and BS-Seq data can be visualized in a single window with a genome viewer. In the screenshot, BS-Seq tracks are included for a segment of chromosome 7 (Chr07, position 25,866,407-25,927,674) showing CG, CHG, and CHH sites for from BS-Seq data, and the fourth track is of MeDIP-Seq peaks. These tracks were viewed via the Integrative Genome Viewer (IGV) program. Chr07 is one of the most highly methylated bean chromosomes, as determined by MeDIP-Seq peak density. Yellow indicates high methylation, while red indicates low or no methylation. **(B)** Screenshot of a segment of chromosome 2 (Chr02, position 24,782,253-24,840,303) via IGV, which is one of the least methylated bean chromosomes, as determined by MeDIP-Seq peak density. Both figures show approximately 60 Kb of sequence at the furthest possible zoom scale to visualize BS-Seq and MeDIP-Seq data in one window.

Since MeDIP-seq is affinity-based and the output is derived from peak shapes, the context of the methylated cytosine is more complicated to determine and less informative. BS-Seq yields individual nucleotide output, therefore, the specific context that is methylated is much more obvious. Because of this difference, precise trends about methylation in specific contexts cannot be made from MeDIP-Seq data. Methylation trends in annotated regions can be examined; for example **Table 2** shows the total peaks, promoter, and genic peaks per chromosome. Genome viewers can be used to compare multiple datasets aligned to the same reference genome, we compared BS-Seq and MeDIP-Seq (**Figures 4A,B**), but other datasets such as RNA-Seq and ChIP-Seq could also be viewed simultaneously (Thorvaldsdottir et al., 2013).

Since researchers are often interested in finding genome-wide differences between a control and treatment, the use of either method would be useful. If only large changes in DNA methylation patterns are of interest, MeDIP-Seq may be the better option, since it is less expensive and uses an affinity-based approach. However, if determining small changes or very specific changes in DNA methylation are desired, BS-Seq is the better option, as it provides much more data. Further, the use of targeted BS-Seq would be the very least expensive per sample. With decreasing sequencing costs, in the future BS-Seq will likely be the best option, as long as there are means to store the data.

## Conclusion

In summary, this is the first genome-wide DNA methylation profiling study in the Meso-American common bean cultivar Sierra using NGS approaches such as BS-Seq and MeDIP-Seq. Methylation statuses of each of the three DNA methylation contexts CG (∼50%), CHG (∼30), and CHH (∼2.7%) were determined from BS-Seq. Overall promoter and genic methylation trends were established. CG was the most commonly methylated context, and was more common in promoters than in genes. The opposite trend was found in CHG and CHH methylation contexts. The majority (93.2%) of MeDIP-Seq peaks were found in intragenic regions.

## Materials and Methods

### Plant Material

The common bean (*P. vulgaris*) cultivar “Sierra,” a pinto bean derived from the Meso-American gene pool was used in this study. Seeds were germinated for 2–3 days, on wet filter paper in petridishes and then planted in 6″ pots, filled with Promix Bx mycorrhizae soil, and grown in a greenhouse, at Delaware State University, Dover, DE, USA. Three separate times, three plants each were grown in the greenhouse under standard conditions, with a photoperiod of approximately 14 h days/10 h nights at 28/20°C (Ayyappan et al., 2015). At 14 days after planting, leaves from the three plants were harvested separately, high quality DNA was extracted, and equal amounts of DNA were combined to represent a single sample from these three plants, called Pool 1. Similarly, the second set of three plants, and the third set of three plants were processed to obtain Pool 1, Pool 2, and Pool 3. Finally, equal amounts of DNA from pools 1, 2, and 3 were combined for the BS-seq experiment. The same sample that was used for BS-seq was also used for the MeDIP-seq experiment so as to help us obtain a direct comparison of these two methods and their efficiency in identifying methylated regions in the genome. Leaves from each plant were collected after two weeks, flash frozen in liquid nitrogen, and stored in a –80°C freezer until DNA was extracted.

### DNA Extraction

DNA was extracted from 2-week old leaves using a CTAB-based protocol as previously described (Doyle, 1990). The quantity and quality of DNA were determined by 1.0% agarose gel electrophoresis and Nanodrop 2000 spectrophotometer (Thermo Scientific, Wilmington, DE, USA). The experiment included a single pooled DNA sample collected from the leaves of nine different plants (biological replicates) and utilized the same sample for generating the MeDIP-Seq and BS-Seq libraries to maintain uniform experimental conditions. Both the libraries were sequenced on the Illumina/HiSeq-2500 platform.

### Methylated Cytosine Sequencing

#### Methyl-MaxiSeq^TM^ Library Construction

Methyl-MaxiSeq^TM^ EpiQuest libraries were prepared from 100 ng of genomic DNA, which was bisulfite-treated using Zymo Research EZ DNA Methylation - Lightning^TM^ kit (Cat#: D5030, Zymo Research, Irvine, CA, USA). The bisulfite conversion rate was determined to be >99%. The bisulfite-converted DNA was subjected to a series of amplifications, which include a primer that contained part of the adapter sequence and four random nucleotides, followed by adding the remaining adapter sequence and barcoding the fragments, respectively. All PCR products were purified using the DNA Clean & Concentrator-5^TM^ (Cat#: D4003, Zymo Research, Irvine, CA, USA). Library fragment size and concentration was checked using the Agilent 2200 TapeStation instrument and sequenced on the Illumina HiSeq 2500 platform (Illumina Inc., San Diego, CA, USA).

#### Methyl-MaxiSeq^TM^ Sequence Alignments and Data Analysis

Sequence reads from bisulfite-treated EpiQuest libraries were identified using standard Illumina base-calling software and then analyzed using a Zymo Research proprietary analysis pipeline written in Python and using Bismark (http://www.bioinformatics.babraham.ac.uk/projects/bismark/) as the alignment software for analysis (Krueger and Andrews, 2011). Index files were constructed by *bismark_genome_preparation* command using the entire reference genome. *Non_directional* and all other default parameters were applied while running Bismark. The methylation level of each sampled cytosine was estimated as the number of reads reporting a C, divided by the total number of reads reporting a C or T. Promoter and gene body annotations were added using *P. vulgaris* genome annotations available at Phytozome (V1.0).

#### MeDIP-Seq Library Construction

Libraries for MeDIP-Seq were prepared following immunoprecipitation using the Zymo Research DNA Methylation IP Kit (Cat #D5101, Zymo Research, Irvine, CA, USA). Immunoprecipitated DNA was subjected to a series of amplifications as described above for preparing bisulfite-converted libraries. All PCR products were purified using the Zymo Research DNA Clean & Concentrator-5^TM^ (Cat#: D4003, Zymo Research, Irvine, CA, USA). The input DNA library was prepared from pooled sample DNA that was fragmented and denatured. Libraries were quantified using the Agilent 2200 TapeStation and by qPCR. Sample concentrations were normalized to 4 nM, and then sequenced on the Illumina HiSeq 2500 platform (Illumina Inc., San Diego, CA, USA).

#### MeDIP-Seq Sequence Alignments and Data Analysis

Sequencing reads were aligned to the reference genome (V1.0) by Bowtie using best mode and other default parameters. Peak calling was done by “MACS2 callpeak” using input DNA as a control. BIGWIG files were generated from the coverage for visualization purposes (Zhang et al., 2008). Sequences derived from MeDIP-seq BS-seq libraries were submitted to the short read archives at NCBI BioProject#PRJNA306503.

## Author Contributions

MC conceived and performed experiments, interpreted the data, and developed the seminal draft of the manuscript. VS performed bioinformatic analysis, interpreted the data, and contributed significantly in drafting the manuscript. KH supervised and contributed to the drafting of the manuscript. VK conceived, supervised the whole project, and contributed to the drafting and editing of the manuscript.

## Conflict of Interest Statement

The authors declare that the research was conducted in the absence of any commercial or financial relationships that could be construed as a potential conflict of interest.

The reviewer JR and handling Editor declared their shared affiliation, and the handling Editor states that the process nevertheless met the standards of a fair and objective review.

## Supplementary Material

**FIGURE S1 | The peak shapes for the input and immunoprecipitated DNA samples are shown.**

**FIGURE S2 | Read coverage across the genome for each methylation context is shown.** The top is CG, middle is CHG, and bottom is CHH contexts. Coverage read ranges from 0 (no coverage), to 1–4 reads, 5–10 reads, 11–20 reads, and 20 or more reads.
