# Supplementary figures and images for: Analyses of Methylomes Derived from Meso-American Common Bean (Phaseolus vulgaris L.) Using MeDIP-Seq and Whole Genome Sodium Bisulfite-Sequencing
Source: Front Plant Sci. 2016 Apr 26;7:447. doi: 10.3389/fpls.2016.00447 (PMC4845718; doi:10.3389/fpls.2016.00447)

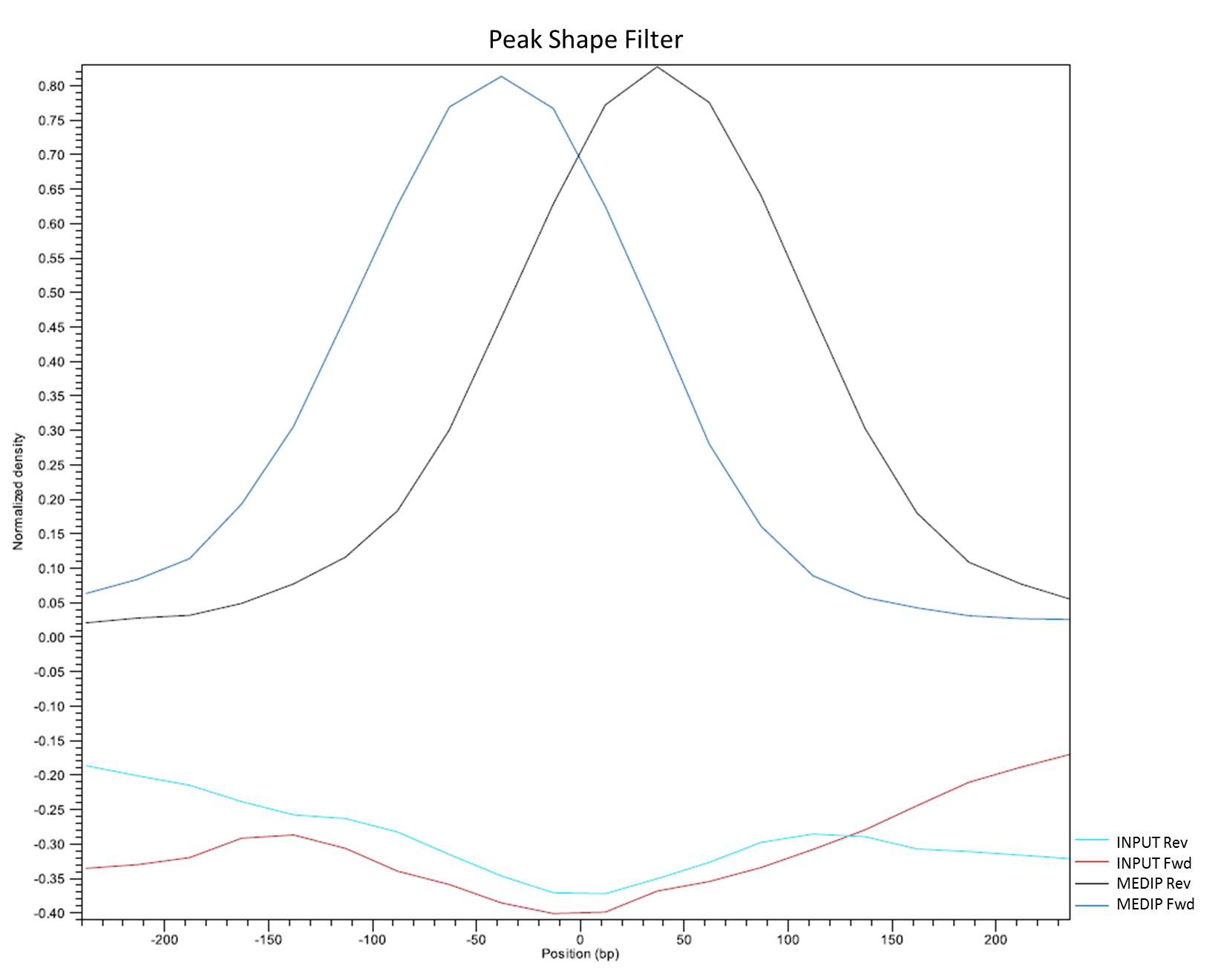

Supplement: Supplementary file 1 [file Image_1.TIFF]

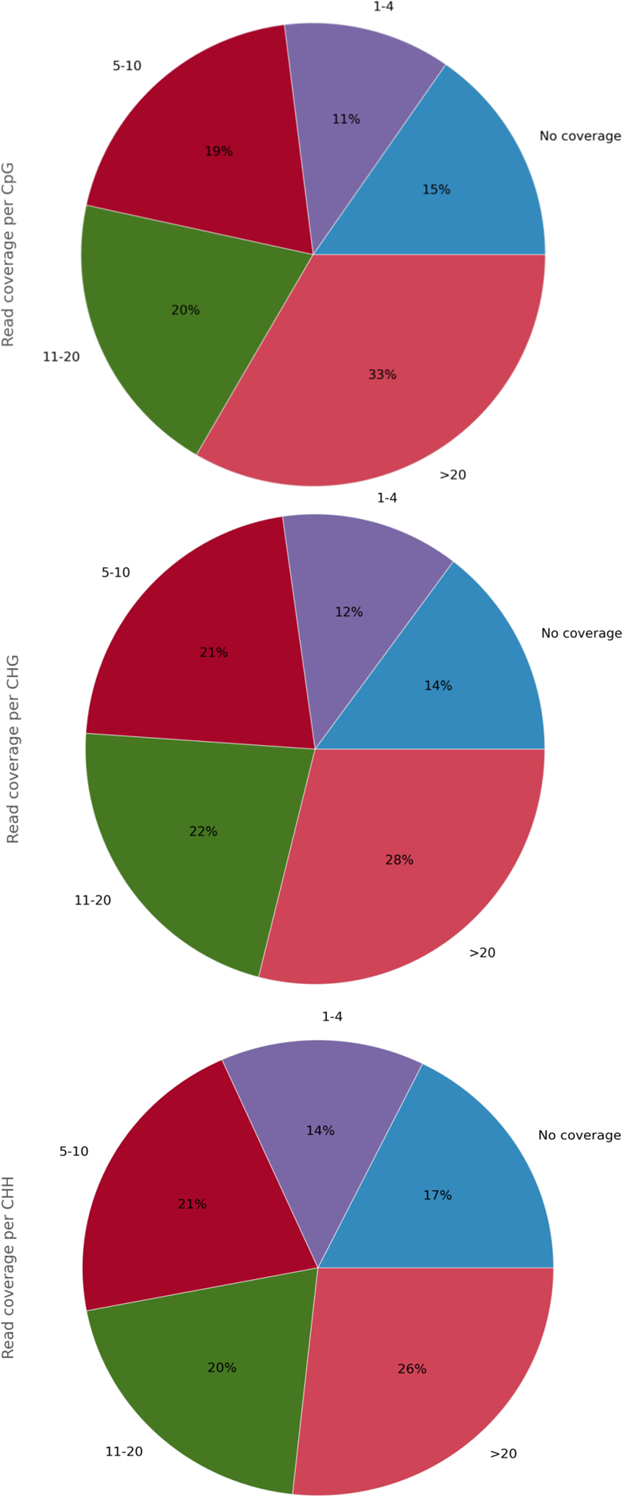

Supplement: Supplementary file 2 [file Image_2.TIFF]
